# A Bidirectional Single‐Cell Migration and Retrieval Chip for Quantitative Study of Dendritic Cell Migration

**DOI:** 10.1002/advs.202204544

**Published:** 2023-01-19

**Authors:** Ning Shao, Yufu Zhou, Jun Yao, Pengchao Zhang, Yanni Song, Kai Zhang, Xin Han, Bin Wang, Xuewu Liu

**Affiliations:** ^1^ Department of Nanomedicine Houston Methodist Research Institute Houston TX 77030 USA; ^2^ The Third Xiangya Hospital Central South University Changsha 410008 P. R. China; ^3^ Department of Molecular and Cellular Oncology The University of Texas MD Anderson Cancer Center Houston TX 77030 USA; ^4^ Department of Breast Surgery Harbin Medical University Cancer Hospital Harbin 150081 P. R. China; ^5^ Department of Genetics The University of Texas MD Anderson Cancer Center Houston TX 77030 USA; ^6^ Present address: Key Laboratory of Advanced Technology for Materials Synthesis and Processing School of Materials Science and Engineering Wuhan University of Technology Wuhan 430070 P. R. China; ^7^ Present address: School of Medicine and Holistic Integrative Medicine Nanjing University of Chinese Medicine Nanjing 210023 P. R. China

**Keywords:** cell retrieval, dendritic cell, microfluidics, RNA‐seq, single cell migration

## Abstract

Dendritic cell (DC) migration is a fundamental step during execution of its adaptive immunity functions. Studying DC migration characteristics is critical for development of DC‐dependent allergy treatments, vaccines, and cancer immunotherapies. Here, a microfluidics‐based single‐cell migration platform is described that enables high‐throughput and precise bidirectional cell migration assays. It also allows selective retrieval of cell subpopulations that have different migratory potentials. Using this microfluidic platform, DC migration is investigated in response to different chemoattractants and inhibitors, quantitatively describe DC migration patterns and retrieve DC subpopulations of different migratory potentials for differential gene expression analysis. This platform opens an avenue for precise characterization of cell migration and potential discovery of therapeutic modulators.

## Graphical abstract

A microfluidic‐based cell migration platform realizes quantitative, high‐throughput, and precise bidirectional migration assays at the single‐cell resolution and controllable retrieval of cell subpopulations with different migratory potential for differential gene expression analysis. This platform has promising potential for precise characterization of cell migration characteristics and evaluation of potential therapeutic modulators.

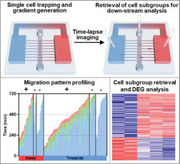

## 1 Introduction

Dendritic cells (DCs) play an essential role in linking innate immunity to adaptive immunity. Immature DCs act as sentinels in peripheral tissues, especially those with abundant antigen exposure (e.g., skin and mucosal surfaces). Upon sampling antigens, they can undergo an activation and maturation process and migrate through afferent lymphatic vessels to draining lymph nodes (LNs). After reaching the draining LNs, DCs present antigens to naive T cells to stimulate lymphocyte expansion and differentiation.^[^ ^1^ ^]^ DC migration is fundamental for execution of their functions during maintenance of immune surveillance and tissue homeostasis, and disease pathogenesis.^[^ ^2^ ^]^ The migration is tightly regulated by a large variety of signals, of which receptor‐chemokine axes have fundamental roles.^[^ ^3^ ^]^ C‐C chemokine receptor 7 (CCR7) and its two ligands, C‐C chemokine ligand 19 (CCL19) and CCL21, form the dominant receptor‐chemokine axis involved in the directional migration of DCs from peripheral sites to lymph nodes.^[^ ^4^ ^]^ Other axes, such as C‐X‐C chemokine ligand 12 (CXCL12) and its receptor C‐X‐C chemokine receptor 4 (CXCR4), and sphingosine‐1‐phosphate (S1P) and S1P receptors, are also involved in this process.^[^ ^5^ ^]^ Better understanding of DC migration is critical for successful manipulation of DC and fine tuning of immune responses in the treatment of allergies and development of efficient vaccines and cancer immunotherapies.^[^ ^6^ ^]^

Traditional approaches for studying cell migration, such as Boyden chambers,^[^ ^7^ ^]^ Dunn and Zigmond chambers,^[^ ^8^ ^]^ and capillary techniques,^[^ ^9^ ^]^ have significant limitations when used to quantitatively and accurately study the diverse patterns and dynamics of cell migration processes. They are either not suitable for time‐lapse imaging of cell migration dynamics, or lack of confinement mimicking in vivo cell migration environment with precise controlling of chemo‐gradient. Microfluidic platforms including microchannels, microchambers, and embedded 3D gel structures are increasingly developed to study cell migration.^[^ ^10^ ^]^ For example, Irimia et al. reported the first microchannel‐based microfluidic device for studying constrained cell migration. They also introduced passive balancing of flow between two fluid streams by contacting them then splitting them again in the migration channel area.^[^ ^10h^ ^]^ Boneschansker et al. introduced a microfluidic platform to simultaneously analyze leukocyte migration signatures both toward and against the chemoattractants by positioning cells in a central channel with two spatial chemo‐gradients on each side.^[^ ^10b^ ^]^ Boribong et al. presented a competitive chemotaxis‐chip that exposed cells randomly in a central channel to competing chemoattractant gradients generated by diffusion between two reservoirs loaded by pipetting.^[^ ^10g^ ^]^ In addition, microfluidic platforms has also been used to study DC migration in vitro and have contributed to significant advances in understanding of DC biology.^[^ ^11^ ^]^ For example, Lennon‐Dumenil's group has been leveraging microchannel‐based microfluidic devices to reveal the underlying mechanisms that regulate DC migration.^[^ ^11a,c–e^ ^]^

While considerable progress has been achieved, many challenges hinder wider use of these platforms in quantitative and accurate study of chemotactic cell migration. In our opinion, an ideal platform for this application would meet the following criteria: it could provide well‐controlled and long‐lasting chemo‐gradient; it could precisely position a large number of cells at the same gradient line at the initiation of the migration and could monitor their migration at the single‐cell resolution; it could mimic the *in‐vivo* cell migration environment such as the confinement and extracellular matrix; it could precisely isolate and retrieve cells with different migratory potentials (i.e., migrating toward chemoattractants, away from chemoattractants and non‐migratory) for downstream analysis. To date, development of platforms that could meet all the above criteria is still a challenge.

Herein we present a bidirectional single‐cell migration chip (BM‐Chip) device with 1000 single‐cell traps and corresponding bidirectional confined migration channels on the two sides of the individual traps. The BM‐Chip assay allows two opposite spatial chemokine gradients on each side of the cell traps, single‐cell resolution, a simultaneous migration start line, and three retrieval sites to retrieve cells migrated toward/against the chemokine source and non‐migratory cells. Using time‐lapse imaging, this platform enables us to examine how the cells sense chemokine gradients, deform and migrate into the confined microchannels and migrate out, as well as the percentages of migrating cells and their directionality. We use BM‐Chip to monitor DC migration in response to different chemoattractants and inhibitors and selectively retrieve cell subgroups with different migratory potentials. The BM‐Chip provides a precise and quantitative platform to investigate migration patterns of single cells and molecular signature of cell subgroups with different migratory potentials, as well as a useful platform for the assessment of anti‐inflammatory agents.

## 2 Results

### 2.1 Principle and Characterization of the BM‐Chip

**Figure 1:**
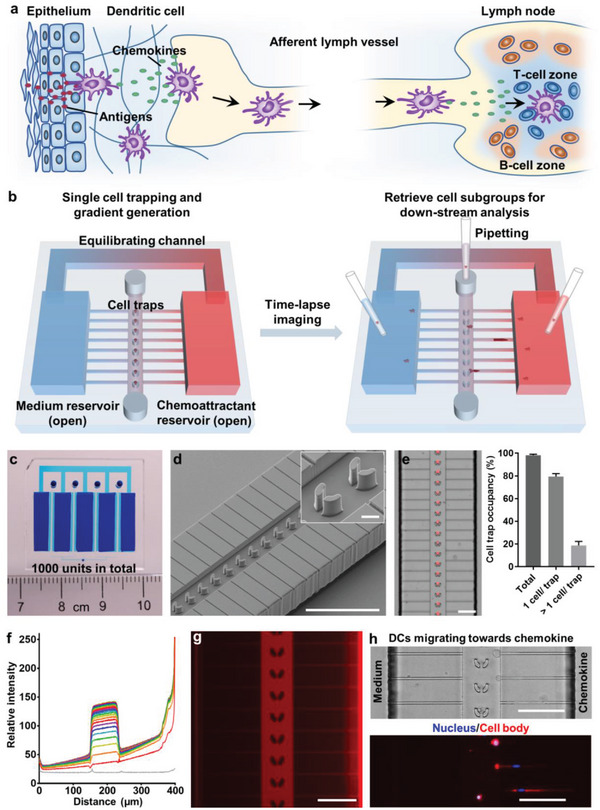
The BM‐Chip is a high‐throughput single‐cell migration assay platform with cell‐retrieval capability. a) Schematic illustration of DC migration to a draining lymph node. b) Schematic view of the BM‐Chip assay from cell loading and gradient generation (left) to cell retrieval after migration (right). c) Image of a BM‐Chip device with reservoirs and channel network indicated by a blue dye. d) A scanning electron microscopy image showing the central cell loading channel with cell trap arrays and the narrow migration channels on each side that connects the reservoirs. Scale bar is 200 µm for the main image and 20 µm for the insert. e) A representative image of the cell trapping and the quantified cell trap occupancies showing generation of single‐cell columns with high efficiency. Data are from four experiments with 500 traps counted in total. Bars represent mean ± standard deviation (SD) values. f) Temporally evolving gradients in the device from 0 min (the gray line on the bottom) to 12 h (the green line on the top) using 10 kDa fluorescein‐labelled dextran as the chemokine‐indicator. Data were collected at the time points from 0 min to 220 min with 10‐min interval, and 4 h, 6 h, and 12 h, individually. The fluorescent signals in the central channel are higher than the signals in the right migration channels because of the different channel heights (20 µm vs 4 µm). g) A representative image of the near‐steady‐state gradients at 210 min. h) Representative images of DC migration in response to a chemokine (CCL19 here). Scale bars are 100 µm for (e) and (g, h).

DC migration from peripheral tissues to afferent lymph vessels and then to draining lymph nodes relies on chemokine guidance (**Figure** **1**a). To mimic DC migration in constrained tissue environments, we designed the BM‐Chip (Figure 1b–d). The BM‐Chip consists of open reservoirs for chemoattractants and media and a connected microfluidic network with three heights: central cell‐loading channels that have a height of 20 µm and contains columns of single‐cell traps, an array of orthogonal confined migration channels that have a height of 4 µm and a width of 5 µm and connect the central cell‐loading channels with the open reservoirs, and large bypass/equilibrating channels that have a height of 150 µm and a width of 1.5 mm and connect the open reservoirs. The dimensions of the structures are presented in Figure S1, Supporting Information. Each chip contains four columns of cell traps (250 cell traps per column) and five large reservoirs all connected to each other with bypass channels. The chip was pre‐functionalized with human collagen type I to mimic the extracellular matrix in vivo. Single cell suspension was loaded into each inlet and flowed into the central channels by negative pressure from the outlet (Figure S2, Supporting Information). Single cells were trapped by the V‐shape hooks, forming uniform and reproducible single‐cell columns with high efficiency (Figure 1e). The width of the central channels, the dimension of the traps and the distance between two neighboring traps have been optimized based on our previous work to achieve high trapping efficiency.^[^ ^12^ ^]^ After loading the cells, the inlets and the outlet was tightly blocked to stop the flow in the central channels. Then the medium in the chemoattractant reservoirs was changed to the medium with the designated chemokine immediately. Our design utilized a design with large bypass channels between the large open reservoirs.^[^ ^13^ ^]^ The large bypass channels rapidly equilibrate the fluid pressure difference between the reservoirs caused by possible uneven fluid levels, and thus prevent convective fluid flow in the migration channels and allows the formation of diffusion‐based gradient. The modelling demonstrated that the bypass channel design could remarkably shorten the fluid pressure equilibrating time for 0.1 mm difference of fluid levels between two reservoirs from 184 h to 173 s (Tables S1, S2, Supporting Information, and calculations in the Supporting Information). We examined the spatial and temporal gradient evolution experimentally by introducing Texas Red‐labeled 10 kDa dextran to the source reservoirs and monitoring the gradient formation by time‐lapse microscopy (Figure 1f,g). Time‐lapse microscopy revealed that a gradient started forming within 10 min and a near‐steady‐state gradient was reached after 210 min. Though the experimental time in this work is 12 h, we demonstrated both experimentally and computationally that the gradient established in the channels was able to sustain for at least 24 h (Figure S3 and Table S3, Supporting Information). It is noteworthy that due to the different heights of the central cell loading channel (20 µm) and the migration channels (4 µm), the imaged fluorescent intensities in the central channel became higher than those in the source side of the migration channels over time. Experiments with cells confirmed that the formed chemokine gradients successfully guided migration of the DCs toward the chemokine CCL19 (Figure 1h). Notably, the elongated cell bodies and nuclei suggest substantial cell confinement and deformation in the microchannels, which also occurs when DCs migrate in narrow interstitial spaces substantially smaller than the cell in cross‐section and when they squeeze through constrictive gaps in the basement membrane to enter lymphatic capillaries.^[^ ^14^ ^]^ After the migration assay, the cells that migrate out to each reservoir can be retrieved by direct pipetting, and the non‐migrating cells that remain in the central channels can be retrieved by reverse‐flushing from the outlet followed by collecting from the inlets using a pipette (Figure 1b). The selective cell retrieval enables following cellular analysis or molecular analysis of gene expression differences between cell subgroups with different chemotactic potentials.

### 2.2 DCs Respond to Different Chemoattractants Differently

**Figure 2:**
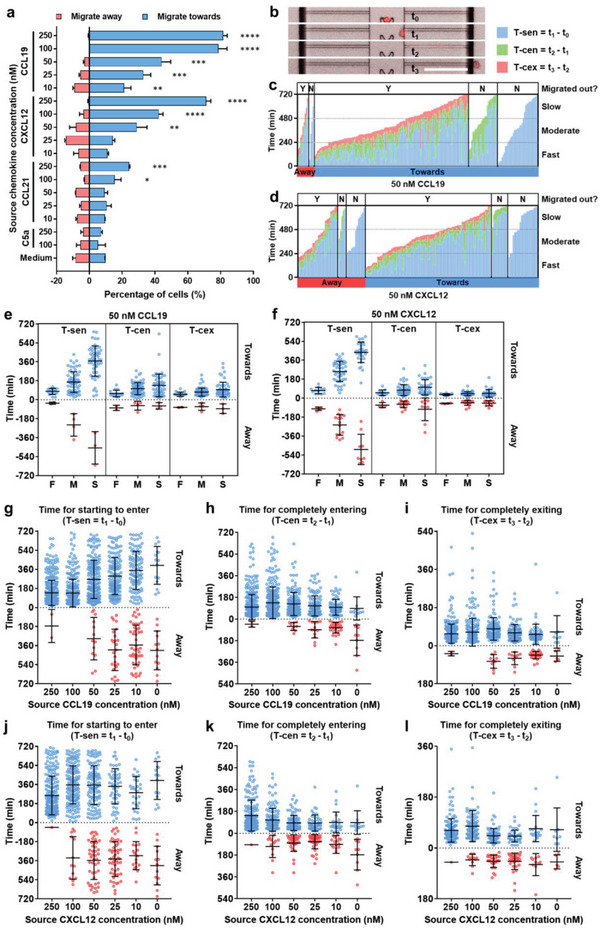
Migration patterns of human mDCs in response to different chemoattractants. a) Percentages of human mDCs migrating toward or away from CCL19, CXCL12, CCL21, and C5a at different concentrations. Percentages of cells migrating toward the chemoattractants were compared with the percentage of cells migrating toward medium. *p < 0.05, **p < 0.01, ***p < 0.001, ****p < 0.0001, Student's *t*‐tests. b) Snapshots showing the four key time points/migration stages for a single DC in the migration assays (i.e., the chemoattractant was added and microscopic recording was started immediately after (*t*_0_), the cell started to enter the migration channel (*t*_1_), completely entered the migration channel (*t*_2_), and completely exited the migration channel (*t*_3_). Scale bar, 100 µm. Migration profiles of migrating human mDCs in response to c) 50 nm CCL19 and d) CXCL12. DC migration was profiled according to their migration directions (away/toward), if they made to each migration stages and the corresponding three lengths of time, that is, the length of time for cells to start to enter the migration channel (T‐sen = *t*_1_ *– t*_0_), to completely entered the migration channel (T‐cen = *t*_2_ *– t*_1_) and to completely exited the migration channel (T‐cex = *t*_3_ *– t*_2_). Each column represents a single migrating cell. Based on their total migrate‐out time, cells were categorized into fast (0−240 min), moderate (245−480 min), and slow (485−720 min) migrators. “Y” and “N” indicate the cells succeeded (Y) or failed (N) in migrating out through the migration channels to the reservoirs, respectively. e,f) Scatterplots of the T‐sen, T‐cen, and T‐cex for fast/F, moderate/M, and slow/S migrators that migrated out, in response to e) 50 nm CCL19 and f) CXCL12. Scatterplots of g) the T‐sen, h) T‐cen, and i) T‐cex for migrating mDCs, in response to different concentrations of CCL19. Scatterplots of j) the T‐sen, k) T‐cen, and l) T‐cex for migrating mDCs, in response to different concentrations of CXCL12. Data are from three independent experiments for (a) and (c–l). The total numbers of recorded cells in time‐lapse imaging in each conditions were between 291 and 523. Bars represent mean ± SD values for (a) and (e–l).

To study DC migration, we first generated DCs from human peripheral blood mononuclear cells (PBMCs) using adherent isolation of monocytes, followed by culturing in medium supplemented with interleukin‐4 (IL‐4) and granulocyte‐macrophage colony‐stimulating factor (GM‐CSF). The induced DCs were then matured using an additional cytokine cocktail and prostaglandin E2 (PGE2). Mature DCs (mDCs) with a distinct surface marker phenotype were characterized using flow cytometry (Figure S4, Supporting Information).^[^ ^15^ ^]^ DC migration is highly sensitive to the geometry of their surrounding environment, in which confinement is a key element. It has been shown that DCs can migrate in 3D environments but not in 2D environments in the absence of integrins, and they migrated significantly faster in confined spaces.^[^ ^16^ ^]^ To compare DC migration in differently confined spaces, we used time‐lapse microscopy to monitor migration of individual DCs in BM‐Chips with different microchannel cross‐sectional dimensions (width×height), that is, 5×1.5 um constrictions, 5×4 µm, 8×5 µm, 16×5 µm, 8×8 µm, and 16×8 µm (Figure S5a, Supporting Information). We found that it took the longest time for DCs to cross the 5×1.5 um constrictions and slightly shorter time to squeeze into the 5×4 µm microchannels (Figure S5b, Supporting Information), which could cause nuclear deformation (Figure 1h). In contrast, it took significantly shorter time for DCs to migrate into the rest four sets of microchannels with bigger sizes and differences among them are minimal. The average migration speeds of DCs in the 5×4 µm microchannels with two 1.5 um constrictions are significantly lower than the migration speeds of DCs in other microchannels, which slightly increased with the increase of channel cross‐section area (Figure S5c, Supporting Information). These data suggest that in confined spaces, nuclear deformation could be a rate‐limiting factor for DC migration if the space is narrower than the size of the cell nucleus. We then characterized patterns of mDC migration in response to four chemoattractants (i.e., CCL19, CCL21, CXCL12, and complement component 5a (C5a)) (**Figure** **2**a). Unlike traditional one‐directional migration assays, the BM‐Chip allowed the cells in the central channel to migrate either toward or away from the chemoattractants. We found that both CCL19 and CXCL12 had strong chemo‐attraction to mDCs. The percentages of DCs migrating toward CCL19 and CXCL12 decreased accordingly as the chemokine concentration decreased gradually from 250 to 10 nm (81.4% to 21.1% for CCL19 and 71.1% to 10.8% for CXCL12) while the percentages of DCs migrating away from the chemokines increased (0.4% to 9.0% for CCL19 and 0.4% to 6.5% for CXCL12). While the chemo‐attraction effect to mDCs was still statistically significant (P<0.01) when the source concentration of CCL19 was as low as 10 nm, the chemoattractant effect was not significant when the CXCL12 concentration decreased to 25 nm. We also found that soluble CCL21 only had moderate chemo‐attraction to mDCs at high concentrations compared to CCL19 and CXCL12 (Figure 2a; Movies S1–S3, Supporting Information). This could be due to the fact that CCL21 guides DC chemotaxis mainly through immobilized gradient in vivo.^[^ ^4b^ ^]^ Though it has been reported that C5a is a potential DC chemoattractant,^[^ ^17^ ^]^ it did not show clear chemo‐attraction to mDCs in our experimental setting. The percentages of cells migrating toward or away from both 250 and 100 nm C5a were roughly equal and were as low as the percentages found under the medium‐only condition. The time‐lapse videos revealed that the cells migrated in the central channel in random directions and only a small number of cells entered the migration channels (Movie S4, Supporting Information).

### 2.3 Quantitative Profiling of mDC Migration in Response to CCL19 and CXCL12

To further characterize the migration patterns of mDCs, we first extracted four key time points during the migration in the BM‐Chip from the time‐lapse microscopy data (Figure 2b). They were: the time that the migration assay was initiated (*t*_0_), the time that a cell sensed the chemo‐gradient and started to enter a migration channel (*t*_1_), completely entered the migration channel (*t*_2_), and completely exited the migration channel (*t*_3_). Based on these four time points, we could calculate the time taken for the DC from assay initiation to starting to enter the migration channel (T‐sen = *t*_1_ − *t*_0_), the time taken from starting to enter to complete entering the migration channel (T‐cen = *t*_2_ − *t*_1_), and the time taken from complete entering to complete exiting the migration channel (T‐cex = *t*_3_ − *t*_2_) (Figure 2b). T‐sen mainly reflects how fast the DC sensed the chemo‐gradient, T‐cen mainly reflects a synergetic effect of both chemo‐attraction and cell deformability and T‐cex mainly reflects how fast the DC migrated in the microchannels. Figures 2c and 2d are representative results showing the migrating status and time patterns of individual migrating DCs in response to 50 nm CCL19 and CXCL12, respectively. The cells were grouped based on their migration directionality and phases. We noticed that although some cells continuously extruded their fronts to the microchannels, they could not completely enter the microchannels. Some of them even died after extensive stretching (Movie S5, Supporting Information). There were also some cells that entered the microchannels but failed to migrate out or died. These phenomena may suggest an important role of cell deformability (including nuclear deformability) in DC migration when they migrate through narrow pores and dense tissues.^[^ ^18^ ^]^ Based on the total time that the DCs took to migrated out the migration channels, we divided the migrated cells into three groups, that is, fast migrator (≤240 min), moderate migrator (245–480 min), and slow migrator (485–720 min). We found that the major difference among the three groups with different migration potential was the time for the cells to start to enter the microchannels (T‐sen) for both chemokines, no matter their migration direction (Figure 2e,f). Slight difference in the time taken to completely enter the microchannels (T‐cen) was also observed among the three groups for the DCs migrated toward the chemokines, but not for the DCs migrated away from the chemokines. Difference in the time taken to completely exit the microchannels (T‐cex) was minimal among the three groups. These results suggests that how fast the DCs sensed the chemo‐gradient and how fast they deformed and squeezed into the confined microchannels majorly determined their total migration time. After they entered the microchannels, they migrated with similar speeds and persistence.

We then applied a series concentrations of CCL19 and CXCL12 from 250 to 10 nm to the BM‐chips and investigated the corresponding DC migration patterns. For CCL19, we found that the higher the chemokine concentration applied, the shorter the T‐sen were for DCs migrating toward CCL19 (Figure 2g). Interestingly, DCs migrating away from CCL19 roughly showed similar trend, although migrating cell numbers were very low when CCL19 concentrations were high. For DCs migrating toward CCL19, the time taken to completely enter the microchannels (T‐cen) and completely exit the migration channels (T‐cex) were similar among different groups (Figure 2h,i). No clear trend was observed on the T‐cen and T‐cex of DCs migrating away from CCL19. This result indicated that after sensing the chemo‐gradients, the DCs migrated with similar speeds and persistence (Figure S6, Supporting Information). We calculated the percentages of total migrating cells and cells migrated out (Figure S7, Supporting Information). Both percentages toward CCL19 decreased significantly with decreased CCL19 concentrations, and the ratios between them were slightly decreased from 84.0% at 250 nm to 63.7% at 10 nm. For CXCL12, the T‐sen of cells migrating toward it increased when the chemokine concentration decreased from 250 to 100 nm, and essentially remained at the same level when the chemokine concentration further decreased (Figure 2j). The T‐sen of cells migrating away from it were also roughly similar (except for the 250 nm‐group in which only one cell migrated away from CXCL12 in three experiments). Similar to the CCL19‐stimulated cells, the T‐cen, T‐cex, and the migration speeds of CXCL12‐stimulated cells were also similar among different groups (Figure 2k,l). The percentages of total migrating cells and cells migrated out toward CXCL12 decreased significantly with decreased CXCL12 concentrations, but the ratios between them did not change significantly (68.0% at 250 nm to 65.6% at 10 nm) (Figure S7, Supporting Information). Taken together, these results indicate that steepness of the soluble chemokine gradient (which is determined by the source concentrations in our experiments here) mainly affects the chemokine‐sensing sensitivity, but has minimal effect on the following cell activities such as cell deformability and motility.

### 2.4 Receptor Inhibition Significantly Diminished mDC Chemotaxis and Reshaped the Migration Patterns

**Figure 3:**
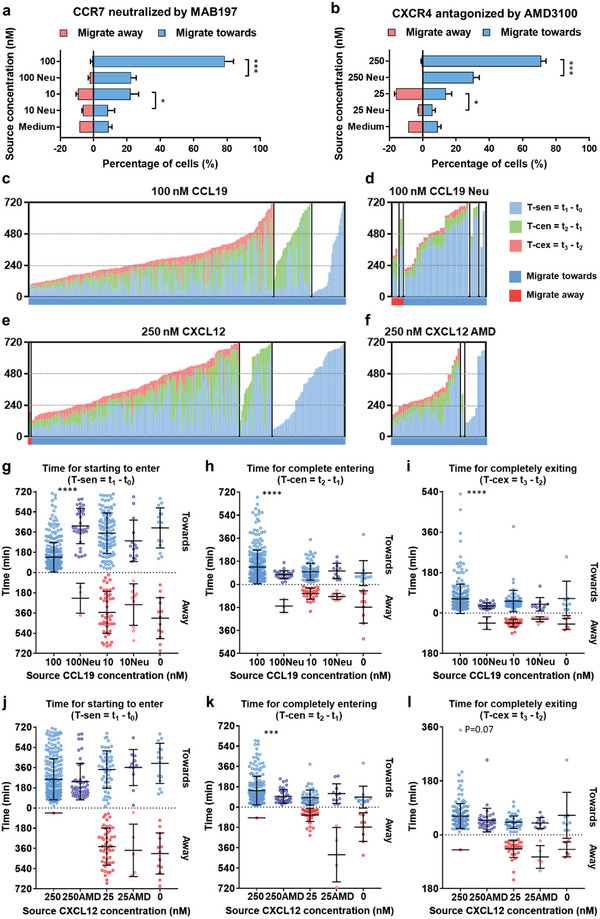
Changes in migration patterns of human mDCs in response to CCL19 and CXCL12 after inhibition of CCR7 and CXCR4, respectively. a) Change of the percentages of mDCs migrating toward or away from CCL19 at 100 and 10 nm after neutralization of CCR7 on mDCs using MAB197 antibody. b) Change of the percentages of mDCs migrating toward or away from CXCL12 at 250 and 25 nm after CXCR4 antagonization by AMD3100. Migration profiles of migrating mDCs in response to CCL19 c) without and d) with CCR7 neutralization. Migration profiles of migrating mDCs in response to CXCL12 e) without and f) with CXCR4 antagonization. Each column represents a single migrating cell for (c–f). Scatterplots of g) the T‐sen, h) T‐cen, and i) T‐cex for migrating mDCs in response to CCL19, after CCR7 neutralization. Scatterplots of the j) T‐sen, k) T‐cen, and l) T‐cex for migrating mDCs in response to CXCL12, after CXCR4 antagonization. Data are from three independent experiments for non‐treated groups in (a–l) and total numbers of loaded cells were between 332 and 523. Data are from two independent experiments for CCR7/CXCR4 inhibited groups in (a–l) and total numbers of recorded cells in time‐lapse imaging were between 144 and 197. Bars represent mean ± SD for (a, b) and (g–l). *p < 0.05, ***p < 0.001, ****p < 0.0001, Student's *t*‐tests for (a, b) and Mann–Whitney tests for (g–l).

Migration of DCs to lymph nodes to initiate adaptive immunity is a critical step in allergic inflammation.^[^ ^19^ ^]^ Inhibition of local DC migration may be a potential route for development of new therapies for allergic inflammation.^[^ ^20^ ^]^ Therefore, we chose a widely used CCR7 neutralizing antibody (MAB197)^[^ ^21^ ^]^ and a FDA approved drug (AMD3100/ plerixafor) that antagonizes CXCR4^[^ ^20^ ^]^ to inhibit DC migration and investigated changes in migration patterns. Treatment with MAB197 (30 µg mL^−1^)^[^ ^21^ ^]^ dramatically diminished the percentages of DCs that migrated toward CCL19 at both 100 nm (from 77.8% to 22.3%, *p* < 0.001) and 10 nm (from 22.0% to 8.4%, *p* < 0.05); the percentages of DCs migrating away from CCL19 did not change significantly (**Figure** **3**a). AMD3100 (1 µg mL^−1^)^[^ ^22b^ ^]^ treatment significantly reduced the fraction of DCs migrating toward CXCL12 at 250 nm, from 71.1% to 30.3% (*p* < 0.001), and at 25 nm, from 13.7% to 5.8% (*p* < 0.05) (Figure 3b). Interestingly, AMD3100 also inhibited cell population migrating away from 25 nm CXCL12, from 15.9% to 2.5%. For both axes, chemokine receptor blocking significantly reduced both the total numbers of migrating cells and numbers of cells that migrated out (Figure S8, Supporting Information). The migrating phases and time patterns of individual migrating DCs in response to CCL19 and CXCL12, without and with chemokine receptor inhibition, were shown in Figure 3c–f and Figure S9, Supporting Information. It is shown that chemokine receptor inhibition reduced all the three populations migrating toward the chemokines, that is, cells not migrated into the microchannels, cells migrated into the microchannels but not migrated out, and cells migrated out. It also reduced all the three populations migrating away from 10 nM CCL19 and 25 nM CXCL12. For the cells successfully migrated out to the chemoattractants, CCR7 neutralization decreased the population of fast and moderate migrators and increased the population of slow migrators. CXCR4 inhibition decreased the population of fast and slow migrators and increased the population of moderate migrators (Figure S10, Supporting Information).

DCs took significantly longer time to sense the CCL19 gradients and to start to enter the migration channels (T‐sen) at 100 nm CCL19 (*p* < 0.0001) (Figure 3g). Surprisingly, migratory CCR7‐neutralized mDCs toward 100 nm CCL19 took less time to completely enter the migration channels and they migrated more quickly through and out the microchannels (statistically shorter T‐cen and T‐cex than the non‐treated group, *p* < 0.0001) (Figure 3h,i). Recent studies have shown that CCR7 uses distinct signaling pathways with a high degree of independence to regulate different mDC migration functions. CCR7‐mediated chemotaxis of mDCs is mainly regulated by the mitogen‐activated protein kinase pathway, while the motility and deformability (migration speed, cytoskeletal rearrangements, actin dynamics, etc.) is mainly regulated by the RhoA‐Rho‐associated protein kinase signaling.^[^ ^20^, ^23^ ^]^ Selective inhibition of each pathway in mDCs did not affect the other pathway and the function associated to it.^[^ ^24^ ^]^ It is highly possible that the CCR7 neutralization only affected the CCL19 sensing, which in turns affected the T‐sen and the percentage of chemotactic cells. The remaining migratory CCR7‐neutralized mDCs might be the selected/enriched migrators that were more sensitive to CCL19 or more potent to activate the downstream pathways that regulate chemotaxis and cell motility. The lower dispersion of the T‐cen and T‐cex values in the CCR7‐neutralized groups also supports the potential selection/enrichment effect. Interestingly, unlike CCR7 neutralization, CXCR4 inhibition by AMD3100 did not delay the time that the migratory DCs sensed the CXCL12 gradient and started to enter the migration channels (T‐sen). We speculate that it could be related to the effect of the other CXCL12 receptor CXCR7.^[^ ^25^ ^]^ It has been reported that CXCL12‐CXCR7 interaction activates ERK1/2 and promotes chemotaxis of Jurkat T cells.^[^ ^26^ ^]^ In addition, AMD3100 is an antagonist of CXCR4 on one hand, it has been found as an allosteric agonist of CXCR7 on the other hand, which positively modulates the effect of CXCL12.^[^ ^27^ ^]^ The time for complete entry into the migration channels (T‐cen) also decreased, which is similar to the CCR7 neutralized group. The migration speed in the microchannels, which is inversely proportional to T‐cex, was not changed significantly. This is in line with the above explanation that chemotaxis of mDCs, defined as “chemoattractant sensing,” and the motility could be different cell activities that are regulated separately. Taken together, these results characterized how receptor inhibition reshapes the migration patterns of mDCs and indicated the complexity of chemotactic signaling. The results may provide new insights into development of therapeutics for allergic inflammation.

### 2.5 Retrieval of mDCs with Different Migratory Potentials for RNA‐Sequencing

**Figure 4:**
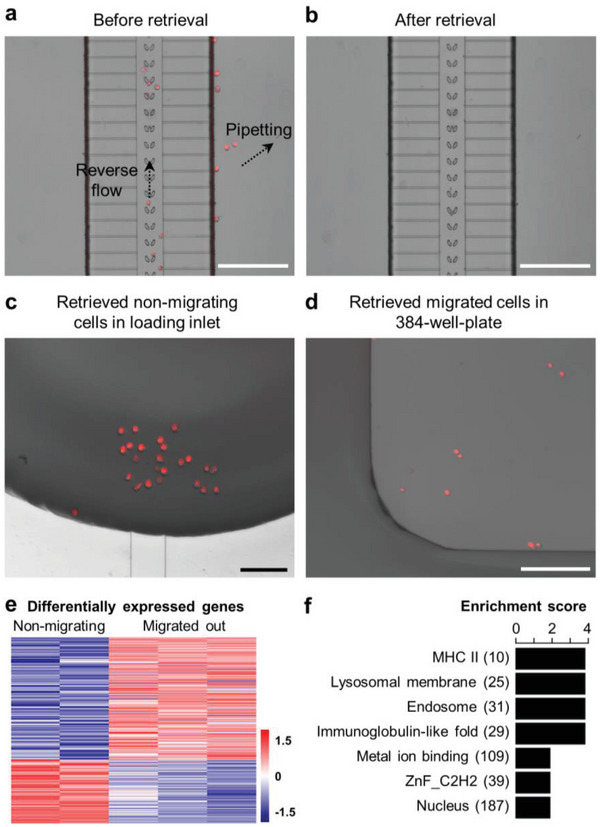
Retrieval of migrated‐out/non‐migrating cells and downstream molecular and cellular analysis. a) Representative image of BM‐Chip before cell retrieval. The migrated‐out mDCs in the reservoirs and the non‐migrating mDCs remained in the central cell‐loading channels were retrieved by direct pipetting or pipetting after reverse flushing, respectively. b) Image of the same area of the BM‐Chip after cell retrieval. Both migrated‐out/non‐migrating cells were successfully retrieved. c) Representative image of non‐migrating mDCs flushed to the inlet. d) Representative image of migrated‐out mDCs retrieved and transferred to a 384‐well‐plate well. Scale bars are 200 µm for (a), (b), (d), and 100 µm for (c). e) Heatmap of differentially expressed genes (DEGs) in migrated‐out and non‐migrating cells from RNA‐seq data analysis. f) Functional annotation of the gene categories enriched in migrated‐out cell DEGs using DAVID analysis.

Precise isolation and retrieval of chemotactically‐different cell subpopulations is of the utmost importance for understanding the fundamental cues of the different chemotactic potential of cells within the same population.^[^ ^10c^ ^]^ To precisely isolate and selectively retrieve cells migrating toward the chemoattractant, away from the chemoattractant, and non‐migrating cells, the same starting line (i.e., the chemo‐gradient) for all the cells to migrate and multiple retrieving sites (i.e., the source reservoirs, sink reservoirs, and cell loading channels) for cell retrieval are required, which most of the existing methods cannot offer. The BM‐Chip allows for cell alignment at the same migration‐starting line and three retrieving sites to isolate and re‐collect cells migrating toward the chemoattractant, away from the chemoattractant, and not migrating. To demonstrate it, we performed a BM‐Chip migration and retrieval assay for mDCs in response to 50 nm CCL19. The cells that migrated to the source reservoirs and sink reservoirs were retrieved by pipetting the medium in the reservoirs and re‐collecting all the medium with the migrated cells in it. The non‐migrating cells that remained in the central channels were gently reverse‐flushed from the outlet and then retrieved from the inlets using a pipette (**Figure** **4**a–d). The retrieval efficiencies for both non‐migrating cells and cells migrated out are both higher than 90% (Figure S11, Supporting Information).

Cell subpopulations isolated and retrieved from the BM‐Chip can be used for downstream molecular or cellular analyses to differentiate underlying between‐population molecular differences or cellular functional differences.^[^ ^28^ ^]^ As a demonstration, we performed RNA‐sequencing (RNA‐seq) for the retrieved mDC subgroups. Because of the extremely low number of cells that migrated to the sink reservoirs, we only collected RNA from the non‐migrating cells and the migrated‐out cells toward CCL19 to ensure adequate RNA materials for sequencing. It is noteworthy that although the amount of retrieved cells migrated to the sink reservoirs was too low to be used in the RNA‐seq, the retrieved cell subgroups with extremely low numbers like in this case could still be used in other types of applications that is compatible with low cell numbers, such as single cell sequencing, cell cloning/proliferation (for other types of proliferating cells but not DCs), etc. Equal numbers of mDCs (approximately 50 cells from each subgroup, three repeats each) were used as the starting material to collect RNA and perform RNA‐seq (Figure 4e,f; Figure S12, Supporting Information). Data from one of the three non‐migrating cell samples was excluded in differential gene expression analysis due to low quality of the sequencing data (Figure S12b, Supporting Information). We identified 704 genes highly expressed in migrated‐out mDCs and 367 genes highly expressed in non‐migrating mDCs (fold change > 2, adjusted *p*‐value < 0.05) (Figure 4e; Dataset S1, Supporting Information). Functional gene annotation was performed on differentially expressed genes enriched in migrated‐out cells using DAVID (the Database for Annotation, Visualization, and Integrated Discover).^[^ ^29^ ^]^ This revealed that migrated‐out cells have enriched genes related to major histocompatibility complex class II (MHC II), lysosomal membrane, endosome, metal ion binding, ZnF_C2H2, and nucleus functions (Figure 4f). Nuclear envelop could rupture when cells migrating through confined spaces such as confined microchannels in vitro and dense tissue and tumors in vivo.^[^ ^30^ ^]^ Rapid and efficient repair of the rupture is critical for maintenance of the cell viability and functions.^[^ ^31^ ^]^ We selected a set of genes from the nuclear envelop repair system and performed quantitative real‐time PCR to validate the expression fold change results of the RNA‐seq (Figure S12d, Supporting Information). The two results were consistent, which further supported the reliability of the RNA‐seq results.

## 3 Discussion

DC migration, which runs through DCs' differentiation and development, is the premise for DCs to execute their biological function in vivo. Dysregulation of DC migration may result in or exacerbate autoimmune diseases, infectious diseases, allergy and tumors.^[^ ^2b^ ^]^ Quantitative characterization and manipulation of DC migration could contribute to the development and improvement of DC‐dependent treatments, such as vaccines and immunotherapies.^[^ ^6^ ^]^ However, current cell migration platforms cannot fully satisfy the needs due to technical challenges. Boyden chambers or transwells^[^ ^7^ ^]^ usually monitor one direction of migration, and imaging the cell migration process is challenging because the migration is perpendicular to the imaging plane. Dunn and Zigmond chambers,^[^ ^8^ ^]^ along with the majority of microfluidics‐based microchambers,^[^ ^10^, ^26^ ^]^ where cells migrate on flat surfaces, lack the cell confinement to recapitulate the microenvironmental cues that the cells migrate through tissue spaces in vivo. In addition, cells seeded in the whole chamber are actually not at the same chemo‐gradient line, which may compromise the precision of the quantification of their migration and the following retrieval of cell subgroups if necessary. Embedding cells in 3D gels can also recapitulate the in‐vivo confined microenvironment for cell migration,^[^ ^10a^ ^]^ but the less‐controlled pore sizes, tortuous migration paths, as well as the incapability of cell subgroup retrieval make 3D gels not the best choice for high‐precision characterization of cell migration.^[^ ^10b^ ^]^ Microchannel‐based migration platforms provide the cell confinement when migrating, and more precise measurements.^[^ ^10b,c,f‐h^ ^]^ However, there are currently still some challenges when applied them to quantitative profiling of single cell migration and precise cell subgroups retrieval. Irimia et al. reported the first microchannel‐based microfluidic device for studying constrained cell migration, in which they also presented a design for passive balancing of flow between two fluid streams by contacting them then splitting them again.^[^ ^10h^ ^]^ However, this platform and similar continuous‐flow‐based platforms are usually lack of precise cell positioning for quantitative comparison and precise retrieval of cell subgroups, and are normally not suitable for non‐adherent cells (such as mDCs and lymphocytes) that could be flushed away by the fluid. In addition, long‐term exposure to flow‐introduced shear stress could interfere cell migration behavior. Boneschansker et al. introduced an innovative microfluidic migration platform with cells trapped in a central channel and two spatial chemo‐gradients on each side, enabling monitoring of cell migration both toward and against chemoattractants.^[^ ^10b^ ^]^ However, the design of closed chemokine reservoirs precludes the retrieval of migrated cells from the reservoirs, thus not suitable for downstream analysis. In addition, although cells were trapped within the central channel, multiple cells from different traps had to share the same channel to migrate, which could interfere each other's migration patterns. Boribong et al. presented a neutrophil chemotaxis‐chip that exposed cells randomly loaded in a wide central channel to competing‐chemo‐gradients generated by perfusion between two open reservoirs loaded by pipetting.^[^ ^10g^ ^]^ Although the open reservoir design simplifies assay operation and may enable subsequent retrieval of migrated cells (not shown in the paper), cells are randomly loaded into a relatively wide central channel before migrating, not forming a single line along the isogradient line, and many cells may compete to migrate through the same channel due to the high cell‐number‐to‐channel‐number ratio, which compromise the precision for quantitative investigation and potential cell retrieval. In addition, two‐reservoir‐based chemotaxis devices without a design to equilibrate possible uneven pressure between the two open reservoirs could easily result in convection flow through the migration channels and disturb the gradient.

By analyzing and comparing the technological merits and drawbacks of different microchannel‐based designs, we designed the BM‐Chip for high‐throughput, precise and bidirectional single‐cell migration assays. Our BM‐Chip platform has several advantages over the existing approaches used for cell migration study. First, BM‐Chip could provide more precise control of initial cell position, besides the simple generation of uniform and long‐lasting chemo‐gradients. The position‐ and gradient‐uniformity could result in more precise and quantitative investigation of cell migration signatures and more precise isolation of cell subpopulations based on migratory potential. Second, BM‐Chip could reliably capture and position as many as 1000 single cells in parallel next to the migration channels. This advantage resulted in single cell resolution and high throughput and avoided interference among cells during migration. Third, BM‐Chip could enable precise isolation and easy retrieval of up to three distinct cell subpopulations, based on their migratory potential. The retrieved cell subpopulations can be used for selection of chemotactically sensitive cells or molecular analysis to link different migratory phenotypes/migration patterns with genotypes.

Our quantitative characterization of DC migration revealed that the concentration of chemoattractant mainly affected the chemo‐gradient sensing, but have minimal influence on the following cell motility related functions. We also found that inhibition of CCR7, the major chemokine receptor for DC migration, mainly inhibited the CCL19‐gradient sensing, but did not significantly affect the following cell deformability and the migration speed inside the confined microchannels. The passively‐selected migrators that overcame the receptor (CCR7/CXCR4) blocking were even faster in migrating into and out the microchannels. These findings highlight a high degree of independence of the pathways that control chemotaxis/chemokine sensing and cell motility. The molecular mechanisms underlying the independence along with the coordination of these pathways, and whether it also applies to other types of immune cells remain elusive. Further investigation of the mechanisms may lead to new development strategies for DC‐related therapies. The precise isolation and retrieval of chemotactically different cell subpopulations using BM‐Chip could also be useful for the mechanism study. Even more precise isolation and retrieval of fast, moderate and slow migrators migrating either toward or against chemoattractant remains a challenge, which requires more advanced technologies. In addition, our RNA‐seq data from chemotactically different mDCs could be further investigated experimentally to explore the molecular mechanisms under the difference in migratory potential. For example, CHMP7 and BANF1 are two genes that both highly expressed in migrated‐out cells, and their encoded proteins have been reported as important elements in repairing of nuclear envelop rupture due to exposure to mechanical force including substantial confinement when migrating.^[^ ^32^ ^]^ Although we couldn't manipulate the expression of these two genes in human mDCs without interfering their migration activity, our preliminary study using a highly metastatic breast cancer cell line MDA‐MB‐231‐BR showed that silencing of either CHMP7 or BANF1 affected the microfluidic‐constriction‐passing rates and time of the cancer cells, and silencing of both genes shows a synergic effect. Since several types of immune cells including DCs always migrate in dense tissue and bear nuclear deformation, we speculate that the two genes may also have important functions in migration of these immune cells, which needs further experimental investigation. Although we only demonstrated the application of BM‐Chip in studying DC migration, we believe that BM‐Chip could also be applied to studying migration of other cell types, including other immune cells, tumor cells and neural cells by adjusting the cell‐trap sizes, migration channel dimensions and chemoattractants.

In summary, we developed a microfluidics‐based cell migration platform that enables high‐throughput and precise bidirectional migration assays and controllable retrieval of cell subpopulations with different migratory potential. We characterized mDC migration signatures in response to different chemoattractants and chemokine receptor inhibitors. Our quantitative investigation brought new insights to the understanding of DC migration, which may be useful for discovery of therapeutic immune modulators. We believe that BM‐Chip could be widely applied to studying cell migration in a more precise manner. BM‐Chip could also be expanded to reveal underlying mechanisms of cell movement and metastasis.

## 4 Experimental Section

### DC Generation and Maturation

DCs were generated and matured from human PBMCs as previously described.^[^ ^14^ ^]^ Briefly, PBMCs (STEMCELL Technologies, MA) were suspended in AIM‐V medium (Thermo Fisher Scientific, MA) at 0.5–1 × 10^7^ mL^−1^ and transferred to tissue culture flasks. PBMCs were incubated at 37 °C in a humidified incubator with 5% CO_2_ for 1.5–2 h to allow for monocyte precursor adherence. All media with suspended cells were then carefully removed using a pipette. Each flask was carefully washed with AIM‐V medium (Thermo Fisher Scientific, MA) to thoroughly remove suspended cells. The monocytes attached to the flask were then cultured in AIM‐V medium with 800 U mL^−1^ GM‐CSF and 500 U mL^−1^ IL‐4 (R&D Systems, MN) in a humidified incubator at 37 °C and 5% CO_2_ for 7 days. The GM‐CSF and IL‐4 were refreshed every 2 days. After 7 days of culture, the generated immature DCs were matured using culture in AIM‐V medium with a cytokine cocktail including GM‐CSF (800 U mL^−1^), IL‐4 (500 U mL^−1^), tumor necrosis factor‐*α* (5 ng mL^−1^), IL‐1*β* (5 ng mL^−1^), IL‐6 (150 ng mL^−1^), and PGE2 (1 µg mL^−1^) (R&D Systems, MN) in a humidified incubator at 37 °C and 5% CO_2_ for 24 h. Mature DCs were harvested by pipetting and washing with ice‐cold medium, then concentrated using centrifugation, resuspended at 5–10 × 10^5^ mL^−1^ in 90% human serum (Thermo Fisher Scientific, MA) and 10% DMSO, divided into aliquots, and frozen. Characterization of mature DCs was performed by flow cytometry analysis using antibodies against human MHC II, CD11c, CD14, CD80, CD86, and CCR7 (BD, NJ). The representative mature DCs phenotypes were MHC II^+^, CD11c^+^, CD14^−^, CD83^high^, CD86^high^, and CCR7^high^.

### BM‐Chip Design and Fabrication

The BM‐Chip was designed using AutoCAD software, printed out as chrome photomasks (Photo Sciences Inc.), and fabricated using multi‐layer photolithography and elastomer molding. Briefly, SU‐8 3005 photoresists (MicroChem Corp.) were spin‐coated onto a 100 mm silicon wafer (Silicon Quest International Inc.) at 4000 rpm for 1 min (Laurell Technologies Corp.) to form a 4‐µm thick film. After soft baking at 95 °C for 1 min, the spin‐coated wafer was exposed to ultraviolet light with the first photomask at an exposure dose of 150 mJ cm^−2^ for 3 s to pattern the migration channels. The wafer was then baked at 95 °C for 1 min and then developed. The second layer of photoresist (SU‐8 3025) was spun at 3000 rpm for 1 min to yield feature heights of approximately 20 µm. After soft baking at 95 °C for 3 min, the wafer was then ultraviolet‐exposed through the second chrome mask for 6 s to generate the central channel and cell trap patterns. Following baking and developing, the third layer of photoresist (SU‐8 2100) was spun at 1500 rpm for 1 min to yield feature heights of approximately 150 µm. After soft baking, the wafer was ultraviolet‐exposed through the third chrome mask for 20 s to generate the reservoir and equilibrating channel patterns. Following baking and development, a final exposure for 20 s and hard baking at 105 °C for 30 min was performed to firm the SU‐8 mold. The mold was silanized for 1 h in a vacuum chamber saturated with trichloromethylsilane vapor (Sigma‐Aldrich) and served as the template for making polydimethylsiloxane (PDMS) devices. The devices were made by pouring the PDMS mixture (10A:1B; Sylgard 184 kit, Dow Corning Corp.) over the master wafer, followed by degassing and curing at 80 °C for 30 min. Then, the PDMS replica was peeled off, cut to the appropriate size, and the inlets and outlets were punched out. The reservoirs were cut out using a blade along the corresponding pattern edges. The devices were plasma‐bonded to standard glass slides and placed in petri dishes. All assembled devices were kept in a vacuum container for at least 30 min before use to avoid bubbles during reagent loading.

### Microfluidic Setup for Dendritic Cell Migration Assay

The device was first filled with 100 µg mL^−1^ human collagen I and incubated at 4 °C overnight, followed by PBS washing and rinsing with AIM‐V medium. Mature DCs were cultured in AIM‐V medium overnight and stained with 1 µm CellTracker Red CMTPX (Thermo Fisher Scientific, MA) for 12 min before loading into the device. After staining and washing, the DCs were resuspended at 5 × 10^6^ mL^−1^ for loading. DC suspensions (2 µL) were added to each inlet and introduced into the central channels using negative pressure generated from a 1‐mL syringe connected to the outlet by microtubing. The dimensions of the central channels and cell traps were carefully designed and optimized to ensure high single‐cell trapping efficiency.^[^ ^12^ ^]^ After capture, free cells were washed away by maintaining negative pressure and pipette‐washing the inlets with culture medium three times. The syringe was then disconnected, and the inlets and outlet were sealed using epoxy glue (Loctite, OH). After waiting 5 min for the glue to solidify, the open reservoirs were refilled with AIM‐V medium. To generate chemo‐gradients in the migration channels, the medium in the designated reservoirs was discarded and immediately replaced with medium with a chemoattractant at desired concentrations. The device was then placed under an EVOS FL Auto Imaging System (Thermo Fisher Scientific, MA) in a humidified incubator at 37 °C and 5% CO_2_ for time‐lapse imaging. Two‐channel (Trans/Texas Red) time‐lapse images from up to 16 areas/beacon over the whole chip were recorded at 5‐min intervals for 12 h (10× objective and automated stage). It was found that the human monocyte‐derived DCs were very sensitive to phototoxicity. Therefore, staining with low dye concentrations and excitation with low light intensity was very important to preserve DC viability and migration capabilities.

### Dendritic Cell Migration Analysis

Time‐lapse images of each beacon were stacked and hyper‐stacked using ImageJ software (National Institutes of Health). Each cell's movement was tracked manually, frame‐by‐frame. Cell number before migration was counted from the first frame. The numbers of cells that migrated toward or away from the chemoattractant were counted from the last frame. A cell that moved into the migration channel for at least a 20‐µm distance was counted as a migrating cell. The direction of migration was recorded for each cell from the last frame. The percentages of cells migrating toward or away from the chemoattractant were calculated from the total numbers of cells migrating toward or away from the chemoattractant, respectively, divided by the total number of cells counted before migration. The percentages of migrating cells or cells migrated out were calculated from the total numbers of migrating cells or cells migrated into the reservoirs, respectively, divided by the total number of cells counted before migration. The time points at which a cell's front edge entered the migration channel (*t*_1_), the entire cell just entered the migration channel (*t*_2_), and the entire cell just exited the migration channel (*t*_3_) were recorded for migrating cells. T‐sen was equal to *t*_1_, T‐cen was calculated by subtracting *t*_1_ from *t*_2_, and T‐cex was calculated by subtracting *t*_2_ from *t*_3_. The overall migration speed was calculated by dividing the migration distance (200 µm) by *t*_3_. The migration speed in the migration channels was calculated by dividing the length of the migration channel (160 µm) by T‐cex.

### Receptor Inhibition Assay

Mature DCs were cultured overnight, then treated with 30 µg mL^−1^ MAB197 (R&D Systems, MN) or 1 µg mL^−1^AMD 3100 (Millipore Sigma, MO) in a humidified incubator at 37 °C and 5% CO_2_ for 2 h. The DCs were kept in the same medium with the inhibitor for loading into the chips. The chips were also prefilled with medium containing 30 µg mL^−1^ MAB197 or 1 µg mL^−1^ AMD 3100. After the DCs were loaded as described above, the inlets and outlet were sealed and 100/10 nm CCL19 or 250/25 nm CXCL12 (along with 30 µg mL^−1^ MAB197 or 1 µg mL^−1^ AMD 3100, respectively, depending on the treatment group) were introduced into the designated reservoirs. Time‐lapse imaging was performed as described above.

### Dendritic Cell Retrieval

After the migration assay, the device was transferred from the incubator to a sterile biosafety cabinet. To retrieve the DCs that migrated into the reservoirs, the media in the open reservoirs with or without chemoattractant was pipetted to suspend the DCs that migrated out and then quickly collected into separate Eppendorf tubes. To retrieve the non‐migrating DCs in the central channels, the seals on the inlets and outlet were removed using a tweezer, followed by gently reverse‐flushing the central channels from the outlet with medium using a microtubing‐connected 1‐mL syringe. The flow rate was kept to <2 µL min^−1^ to avoid damage to the cells. The cells that flowed out into the inlets were then suspended in 5 µL medium and collected into an Eppendorf tube. All cell subpopulations retrieved in Eppendorf tubes were washed one time with PBS and were used for downstream molecular analysis.

### RNA‐Seq and Data Processing

The RNA‐seq was performed as previously described.^[^ ^28c^ ^]^ Equal numbers of cells (50 per sample) were used as starting material for migrating/non‐migrating groups. All subsequent procedures were performed in a clean and DNase/RNase‐free working station to avoid contamination and DNA/RNA degradation. Briefly, cDNA was synthesized and amplified using the SMART‐Seq v4 Ultra Low Input RNA Kit (Takara Bio USA), following the manufacturer's instructions. After purification using Agencourt AMPure XP beads (Beckman Coulter, IN), the cDNA was quantified using a High Sensitivity DNA Chip (Agilent, CA) and an Agilent 2100 Bioanalyzer. RNA‐seq libraries were generated from cDNA using the Illumina Nextera XT DNA Sample Preparation Kit. Quality of the cDNA library was assessed using an Agilent 2100 Bioanalyzer. RNA‐Seq was performed on the Illumina HiSeq4000 platform in the 150 bp pair‐end configuration, using a service from LC Sciences (Houston, TX, USA). Raw sequencing reads in fastq format were mapped to human transcriptome hg38 using Salmon (v0.14.1) which generated raw gene counts as well as normalized transcript per million (TPM) values. DEG analysis was performed using DESeq2 followed by a filtering step to keep genes with more than twofold changes, *p* < 0.05, and a median log2(TPM) value higher than −1 in the higher expression group. DAVID analysis was performed on the website https://david.ncifcrf.gov/tools.jsp, using DEGs found in migrated‐out cells. RNA‐seq data is available at the National Center for Biotechnology Information Gene Expression Omnibus, under accession number GSE207620.

### RT‐qPCR Validation

RT‐qPCR was performed using the iTaq Universal SYBR Green Supermix (Bio‐Rad, CA). Briefly, the 10‐µL reaction mixture consisted of 4 ng cDNA, 500 nm primer, and 1× Supermix. The primer sequences are shown in Table S5, Supporting Information. The PCR program was set to 40 cycles of denaturation at 95 °C for 15 s and annealing and extension at 60 °C for 60 s, followed by the default melting curve analysis program. The experiment was performed in triplicate for each gene and GAPDH was used as an endogenous control to normalize each sample. The reactions were performed on the StepOnePlus Real‐Time PCR System (Applied Biosystems, MA). The data were analyzed using the standard 2^(−Delta Ct)^ method to obtain the results for gene expression change.

### Image Acquisition and Analysis

BM‐Chip morphology was imaged using a Nova Nano scanning electron microscopy 230 instrument (SEM, high vacuum, HV = 5 kV). Except for the time‐lapse images, fluorescence images were obtained using an EVOS microscope (Thermo Fisher Scientific, MA). The images were analyzed using ImageJ software (National Institutes of Health).

### Statistical Analysis

The statistical analysis was performed using GraphPad Prism 8 software (GraphPad Software, CA). Unpaired Student's *t*‐tests (two‐tailed) were used to compare datasets with Gaussian distributions, and Mann–Whitney tests were used for datasets without Gaussian distributions. Differences were considered statistically significant when *p*‐values were <0.05. All estimated errors for analysis of biological replicates were SD values (unless otherwise stated in figure legends).

## Conflict of Interest

The authors declare no conflict of interest.

## Supporting information

Supporting Information

Supplemental Movie 1

Supplemental Movie 2

Supplemental Movie 3

Supplemental Movie 4

Supplemental Movie 5

Supplemental Movie 6

Supplemental Dataset 1

Supplemental Table 1‐4

## Data Availability

The data that support the findings of this study are available in the supplementary material of this article.
