# Supplementary material for: A Bidirectional Single‐Cell Migration and Retrieval Chip for Quantitative Study of Dendritic Cell Migration
Source: Adv Sci (Weinh). 2023 Jan 19;10(8):2204544. doi: 10.1002/advs.202204544 (PMC10015900; doi:10.1002/advs.202204544)
Supplement: Supplementary file 1 — Supporting Information [file ADVS-10-2204544-s008.pdf]

ADVANCED SCIENCE
Open Access

Supporting Information

for *Adv. Sci.*, DOI 10.1002/advs.202204544

A Bidirectional Single-Cell Migration and Retrieval Chip for Quantitative Study of Dendritic Cell Migration

Ning Shao, Yufu Zhou, Jun Yao, Pengchao Zhang, Yanni Song, Kai Zhang, Xin Han, Bin Wang* and Xuewu Liu**

Supporting Information

A Bidirectional Single-Cell Migration and Retrieval Chip for Quantitative Study of Dendritic Cell Migration

Ning Shao, Yufu Zhou, Jun Yao, Pengchao Zhang, Yanni Song, Kai Zhang, Xin Han, Bin Wang* and Xuewu Liu**

1. Modelling fluid level-equilibrating efficacy of the bypass channel.

The modelling was based on a method described previously.[1] Briefly, we calculated the time required for equilibrating the fluid level differences between two reservoirs in the absence and presence of a bypass channel, and the fluid flow through the migration channels and the bypass channel in the presence of given fluid level differences. For a laminar flow here with low Reynolds numbers, the volumetric flow rate, Q, through a channel is determined by Equation S1:

$$Q = \frac{\rho g \Delta h}{R} \qquad (1)$$

where ρ is the density of the medium, g is the gravitational acceleration, Δh is the height difference between two reservoirs and R is the overall hydrodynamic resistance of the given microfluidic system. For a microfluidic channel with aspect ratios with $w > h$, the hydrodynamic resistance, R, at low Reynolds numbers is given by the approximate Equation S2:

$$R = \frac{12 l \mu}{w h^3 \left(1 - 0.63 \frac{h}{w}\right)} \qquad (2)$$

where l , w, h and μ are length of the channel, width of the channel, height of the channel, and viscosity of the fluid, respectively. The overall resistance of the BM-Chip with/without the bypass channel can be approximated as a combination of the in-parallel migration channels on one side, the central cell-loading channel, the in-parallel migration channels on the other side in series, and with/without the bypass channel arranged in parallel.

For a number of channels arranged in parallel, the total resistance $R_{total\_parallel}$ is given by Equation S3:

$$\frac{1}{R_{total\_parallel}} = \sum \frac{1}{R_i} \qquad (3)$$

where R_i is the resistance of an individual channel.

For a number of channels arranged in series, the total resistance R_{total_series} is given by Equation S4:

$$R_{total\_series} = \sum R_i \qquad (4)$$

As fluid flows from one reservoir into the other, the height difference Δh over time depends on the exchanged volume, Q, and the cross-sectional area of the reservoir, A, is given by Equation S5:

$$\Delta h(t) = \Delta h_0 e^{-\frac{2\rho g}{AR}t} \qquad (5)$$

where Δh_0 is the initial height difference between the two reservoirs.

Therefore, the time *t* required for equilibrating an initial fluid level difference Δh_0 to a given $\Delta h(t)$ can be calculated by Equation S6:

$$t = -\frac{AR}{2\rho g} ln\left(\frac{\Delta h(t)}{\Delta h_0}\right) \qquad (6)$$

The total exchanged fluid volume over time, *V*, can be determined by Equation S7:

$$V(t) = \int_0^t Q(t) = \frac{\Delta h_0 A}{2}\left(1 - e^{-\frac{2\rho g}{AR}t}\right) \qquad (7)$$

The computing parameters are shown in Table S1.

**Parameter**	**Migration channel**	**Cell-loading channel**	**Bypass channel**
**Channel length [µm]**	160	75	10000
**Channel width [µm]**	5	14000	1500
**Channel height [µm]**	4	20	150
**Number of channels**	250	1	1
**Viscosity [N s/m^2]**	0.00069		
**Hydrodynamic resistance [N s m^{-5}]**	Single migration channel	8.34677×10^{15}	
	One side of migration channels	3.33871×10^{13}	
	Cell-loading channel	5.54964×10^{9}	

	Total migration/ cell-loading channels	6.67797×10^{13}
	Bypass channel	1.74552×10^{10}
	Total with bypass channel	1.74507×10^{10}
**Density [g m^{-1}]**	993.4	
**Gravitational acceleration [m s^{-2}]**	9.8	
**Length of the reservoir [mm]**	14	
**Width of the reservoir [mm]**	3	
**Height of the reservoir [mm]**	3	
**Diffusion constant [m^2 s^{-1}]**	1.42×10^{-10}	
**Initial concentration [g m^{-3}]**	2.2	

Table S1. Computing parameters for the results in Table S2 and Table S3**.**

The computing results are shown in Table S2. The data shows that the equilibration time could be reduced remarkably from hundreds of hours to 3−4 minutes with the bypass channel, avoiding long-term convective fluid flow in the migration channels due to the fluid level difference between reservoirs.

**Equilibration between fluid levels in two reservoirs to 1 µm**	**Initial fluid level difference [mm]**			
	1	0.5	0.25	0.1
**Time *T* to equilibrate levels with bypass channel [s]**	260.0	233.9	207.8	173.4
**Time *T* to equilibrate levels to 1 µm without bypass channel [h]**	276.4	248.7	221.0	184.3
**Excess volume in one reservoir [µL]**	42	21	10.5	4.2
**Volume exchange through bypass channel within Time *T* [µL]**	20.978962	10.478966	5.22897	2.078975
**Volume exchange through migration channels within Time *T* [µL]**	0.037873	0.017038	0.00757	0.002685
**Percentage of migration channel volume displaced within Time *T***	167.40%	75.31%	33.46%	11.87%

Table S2. Fluid level-equilibrating efficacy of the bypass channel.

2. Modelling mass exchange between sink and source reservoirs by the migration channels and the bypass channels over time of diffusion.

To investigate whether diffusion through the migration channels and the large bypass channel could result in rapid equilibration of the concentrations between the source and sink reservoirs, we calculated the mass exchange rate of CCL19 by diffusion through the migration channels and the bypass channel within 12 h and 24 h, using Fick's law/Equation S8.

$$J = -D\frac{\partial C}{\partial x} = \frac{m}{At} \qquad (8)$$

where *J* is the diffusion flux, of which the dimension is amount of substance per unit area per unit time, *d* is the diffusion coefficient or diffusivity of the molecule,[2] *c* is the concentration of the molecule, *x* is the length of the channel of interest, *m* is the mass exchange, *A* is the cross-section area of the channel and *t* is the diffusion time. Using this Equation and a conservative estimate that the concentrations at the sink and source reservoirs remain constant, the mass exchange *m* can be deduced, as shown in Equation S9:

$$m = -D\frac{C}{x}At \qquad (9)$$

The diffusion rate within time can be calculated using Equation S10:

$$\mathrm{m}(\%) = \frac{m_{\_exchanged}}{m_{\_initial}} = \frac{m_{\_exchanged}}{Clwh} \qquad (10)$$

where *c*, *l*, *w*, *h* are the initial concentration of the molecule, the length, width and height of the reservoir, respectively.

The computing parameters are shown in Table S1 and the computing results are shown in Table S3. It is shown that during the 12 hours of experimental time, only 0.15% of the chemoattractant in the source reservoir could be exchanged. After 24 hours, only 0.3% of the chemoattractant diffused across the channels. These results further confirm that diffusion through the migration channels and the large bypass channel could not result in rapid equilibration of the concentrations between the source and the sink reservoirs.

It is noteworthy that the total mass transfer could result from not only the diffusion but also the convection flow from one reservoir to the other one through the bypass channel due to the equilibration of pressures between the two reservoirs (Table S4). To minimize the influence of the convection flow for equilibration on the gradient establishment, one should minimize the initial fluid level difference when adding chemoattractants. Since the reservoirs are always filled up with fluid in practice, it‘s achievable to make the initial fluid level difference lower than 0.1 mm in operation, which makes the mass transfer by the convection flow lower than

1.67% of the total. In addition, using the approximate Equation S11 of diffusion time and distance:

$$x = \sqrt{2TD} \qquad (11)$$

where x, T and D are the diffusion distance, the diffusion time and the diffusion coefficient, respectively, the diffusion distance of the chemoattractant is ~3.5 mm after the 12-h assaying time. This diffusion distance is close to the distance between the opening of the bypass channel and the closest migration channels. This suggests that the diffusion of the tiny amount of chemoattractant exchanged through the bypass channels could not influence the gradient in the majority of the migration channels, which is in accordance with our observation in practice.

**Mass exchange [g]**		**Time duration [h]**	**Mass exchange rate**
**Migration channels**	2.11×10^{-10}	12	0.076%
**Bypass channel**	2.02×10^{-10}	12	0.073%
**Migration channels**	4.22×10^{-10}	24	0.152%
**Bypass channel**	4.05×10^{-10}	24	0.146%

Table S3. Mass exchange between sink and source reservoirs by the migration channels and the bypass channels over time of diffusion.

**Initial fluid level difference [mm]**	1	0.5	0.25	0.1
**Total volume in the reservoir [µL]**	126			
**Excess volume in one reservoir [µL]**	42	21	10.5	4.2
**Volume exchange until equilibration [µL]**	~21	~10.5	~5.25	~2.1
**Mass exchange rate**	~16.67%	~8.33%	~4.17%	~1.67%

Table S4. Mass exchange between sink and source reservoirs by the convection flow through the bypass channels over time of equilibration.

Name	Sequence (5’-3’)
GAPDH-F	ACAACTTTGGTATCGTGGAAGG
GAPDH-R	GCCATCACGCCACAGTTTC
CHMP1B-F	TGGAAGTTGCGAGGATACACG
CHMP1B-R	CGCGCACTCATTCTCAAGAAAT
CHMP3-F	AAGCATGGACGATCAGGAAGA
CHMP3-R	CTGGAAGGGCATCAGTCACTT
CHMP6-F	TTGAGTTCACCCAGATCGAAATG
CHMP6-R	TGGCAGCTCTATTTGTTCCTG
CHMP7-F	AGGAGGTGTATCGTCTGTATCAG
CHMP7-R	TCTCATCTGGGCAGGAGTTAG
BANF1-F	TGGCTGAAAGACACTTGTGG
BANF1-R	CACTCTCGAAGGCATCCGAAG

Table S5. Primer sequences used in RT-qPCR assays.

Supplementary Figures

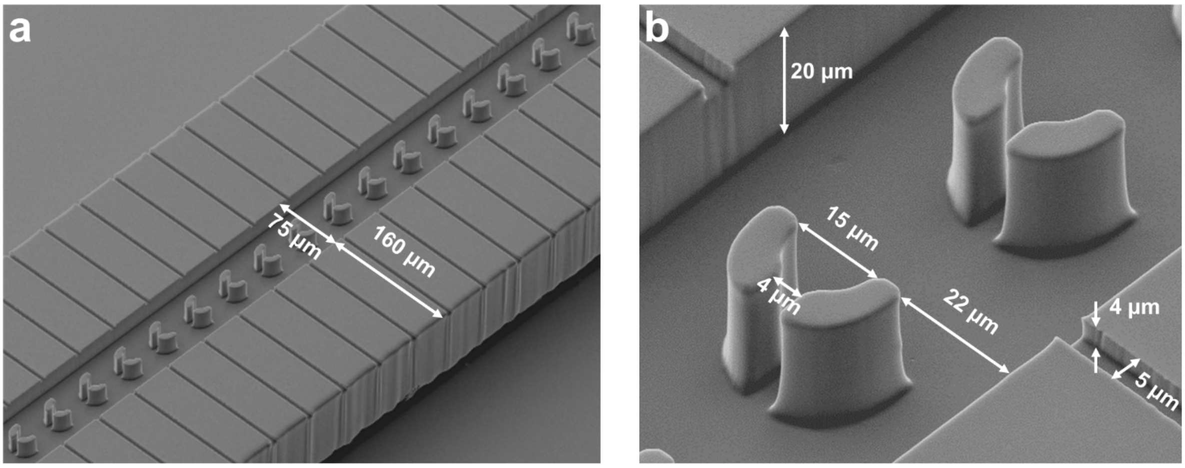

Figure S1. Dimensions of BM-Chip structures. a) Overview of chip morphology. b) Magnified view of single-cell traps and migration channels.

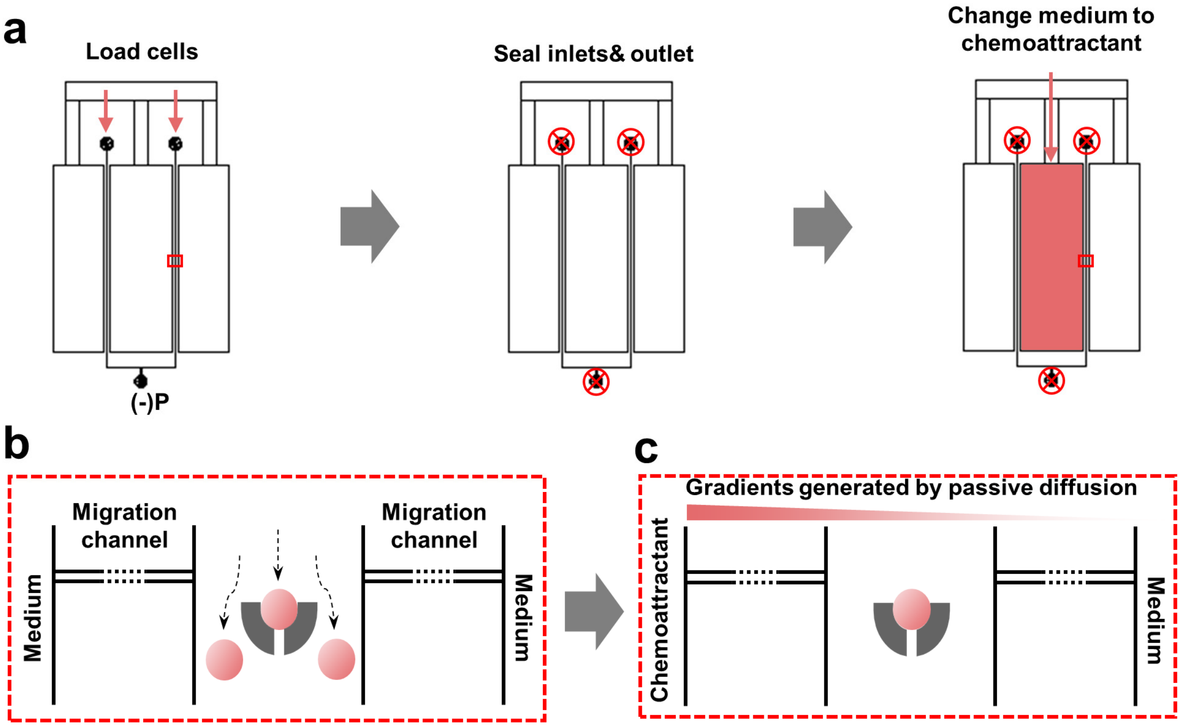

Figure S2. Workflow for single-cell trapping and chemo-gradient generation. a) Workflow for the BM-Chip setup, including cell loading, inlet/outlet sealing, and chemoattractant adding. b,c) The small red boxes in a) are magnified for clearer schematic views of the single-cell trapping (b) and chemo-gradient generation (c).

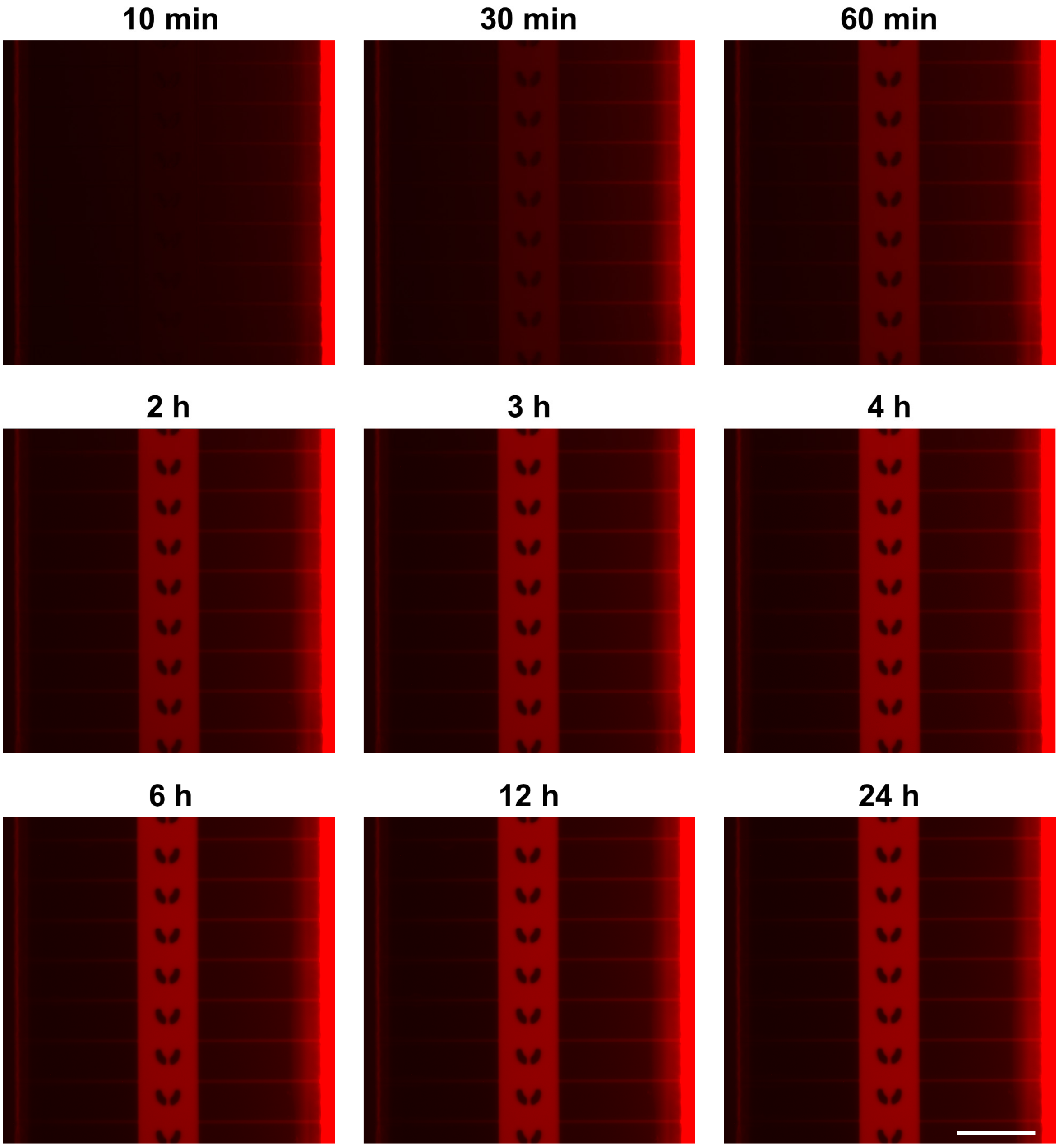

Figure S3. The spatial and temporal gradient evolution indicated by Texas Red-labeled 10 kDa dextran. The gradient started forming within 10 minutes. The gradients from 4 h to 24 h was nearly stable.

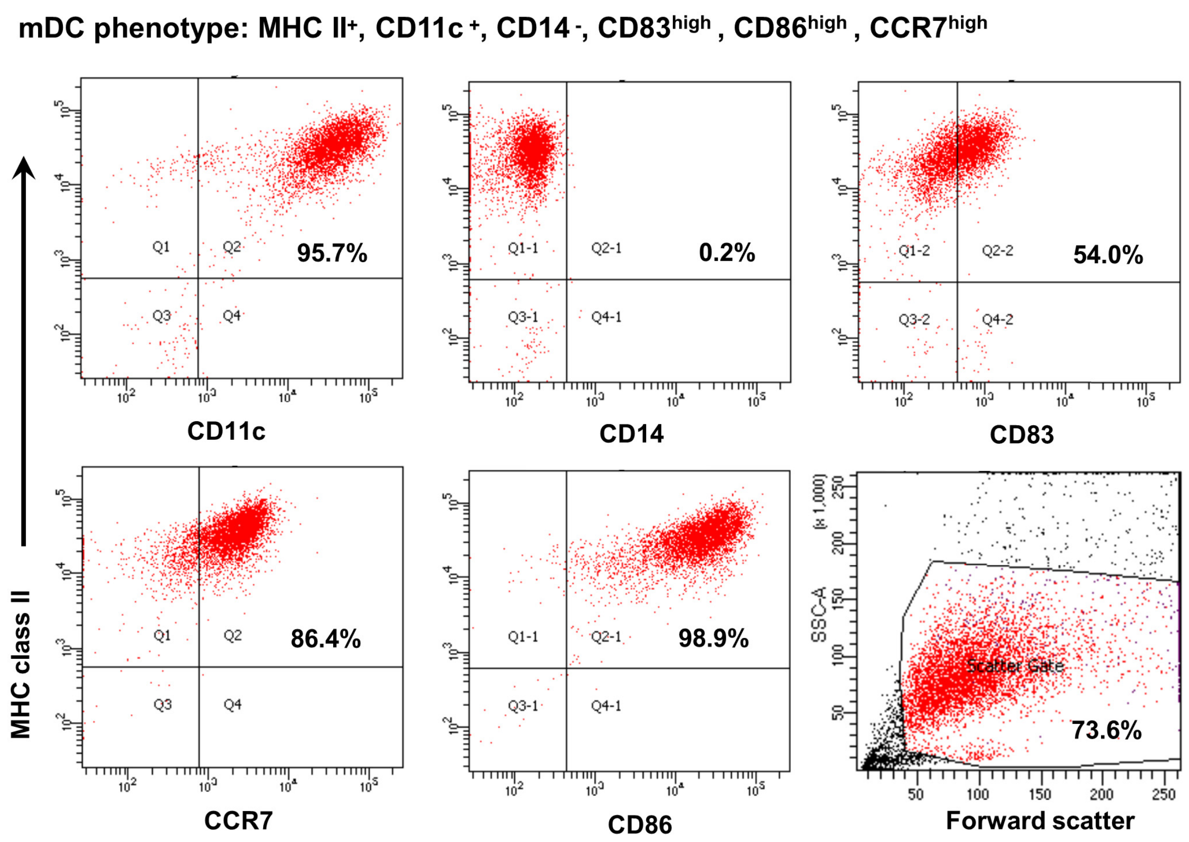

Figure S4. Phenotype and scatter profiles of human monocyte-derived mature DCs characterized by flow cytometry.

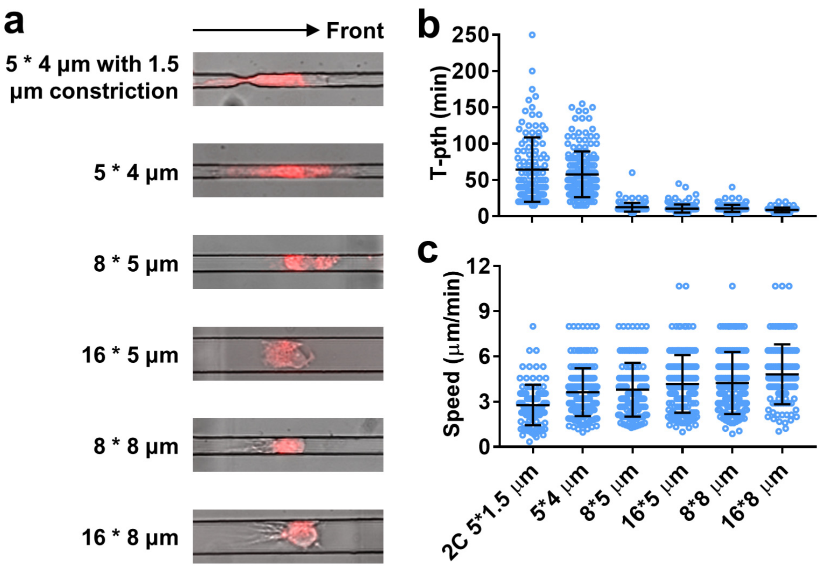

Figure S5. Comparision of chemotactic migration of human mDCs in microchannels with different cross-sectional dimensions in response to CCL19. a) Representative snapshots of the cells migrating in microchannels with different dimensions. All the channel dimensions are labelled as channel width * channel height. b) Scatterplots of the time for the cells to completely pass through microchannel openings or constrictions. T-pth represents the time between the cell front approaching the channel opening/constriction and the cell rear completely passing through the opening/constriction. c) The average migration speeds of cells in the microchannels with different dimensions. "2C 5*1.5 μm": two separate 5*1.5 μm constrictions within a 5*4 μm microchannel. The migration speed was calculated by dividing the length of the microchannel by the length of time between the cell just completely entering and exiting a microchannel. The results represent combined data from two independent experiments for b) and c). Only the cells succeeded in migrating out from the migration channels to the CCL19 reserviors were measured. The total numbers of measured cells in each conditions were between 113 and 171. Bars represent mean ± standard deviation (SD).

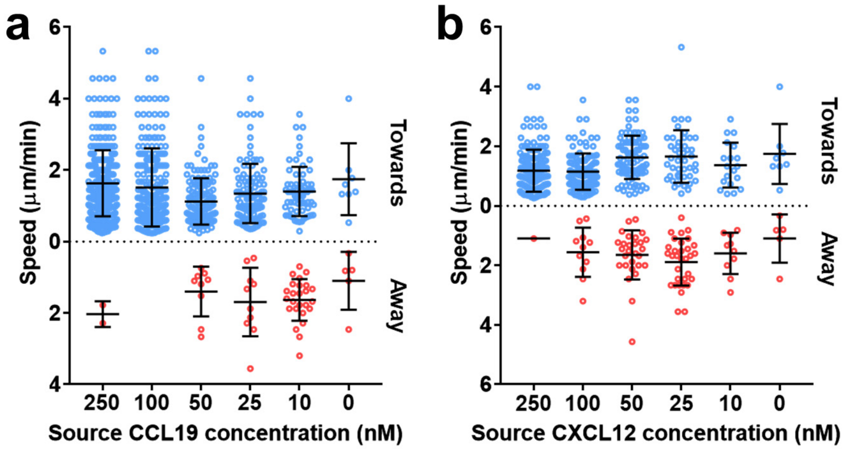

Figure S6. Scatterplots of the overall migration speeds of mDCs in response to CCL19 (a) and CXCL12 (b). The speed was calculated by dividing the length of the migration channel by the length of time between the cell front approaching the migration channel and the cell rear migrated out the migration channel. The results represent combined data from three independent experiments. The total numbers of recorded cells in time-lapse imaging in each conditions were between 291 and 523. Bars represent mean ± standard deviation (SD).

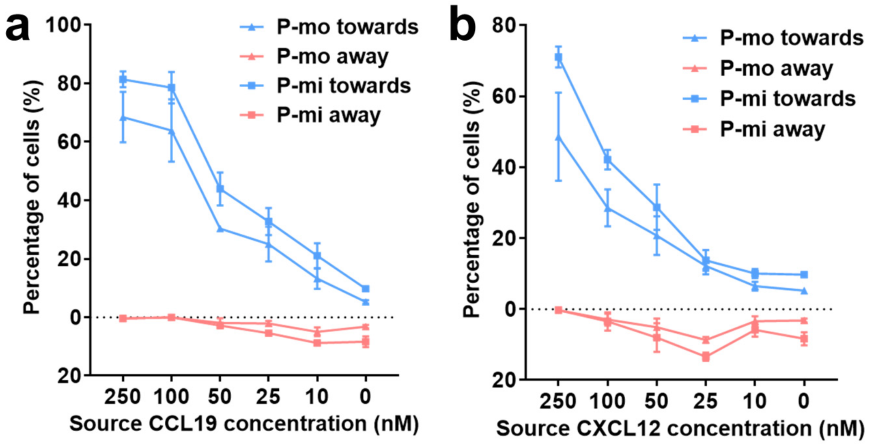

Figure S7. Percentages of total migrating (P-mi) and migrated-out (P-mo) mDCs in response to CCL19 (a) and CXCL12 (b) at different concentrations. All results represent combined data from three independent experiments. Bars represent mean ± SD.

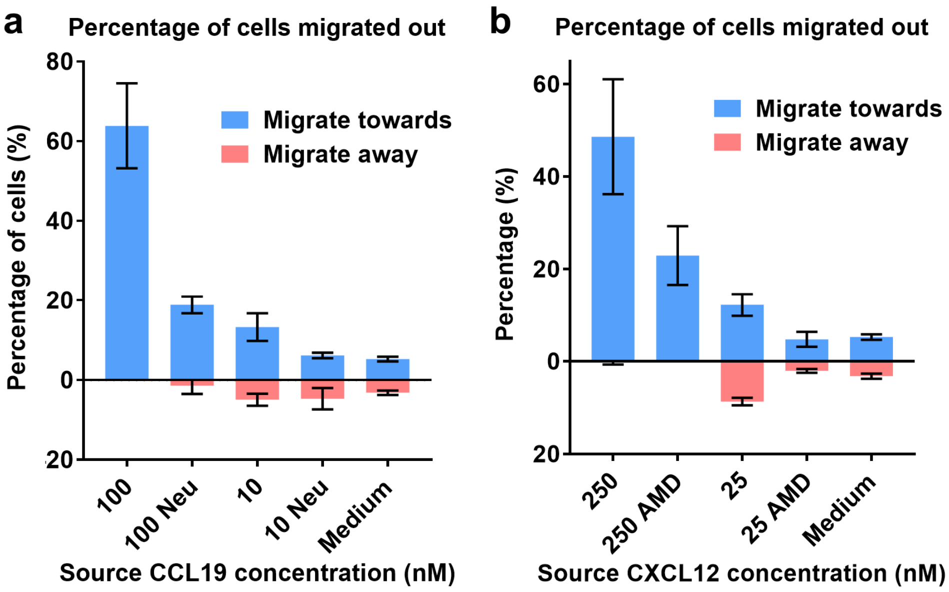

Figure S8. Changes of the percentages of the cells migrated out in response to 100 nM and 10 nM CCL19 after CCR7 neutralization by MAB197 (a) and in response to 250 nM and 25 nM CXCL12 after CXCR4 antagonization by AMD3100 (b). Results represent combined data from three independent experiments for non-treated groups and are from two independent experiments for CCR7/CXCR4 inhibited groups. Bars represent mean ± SD.

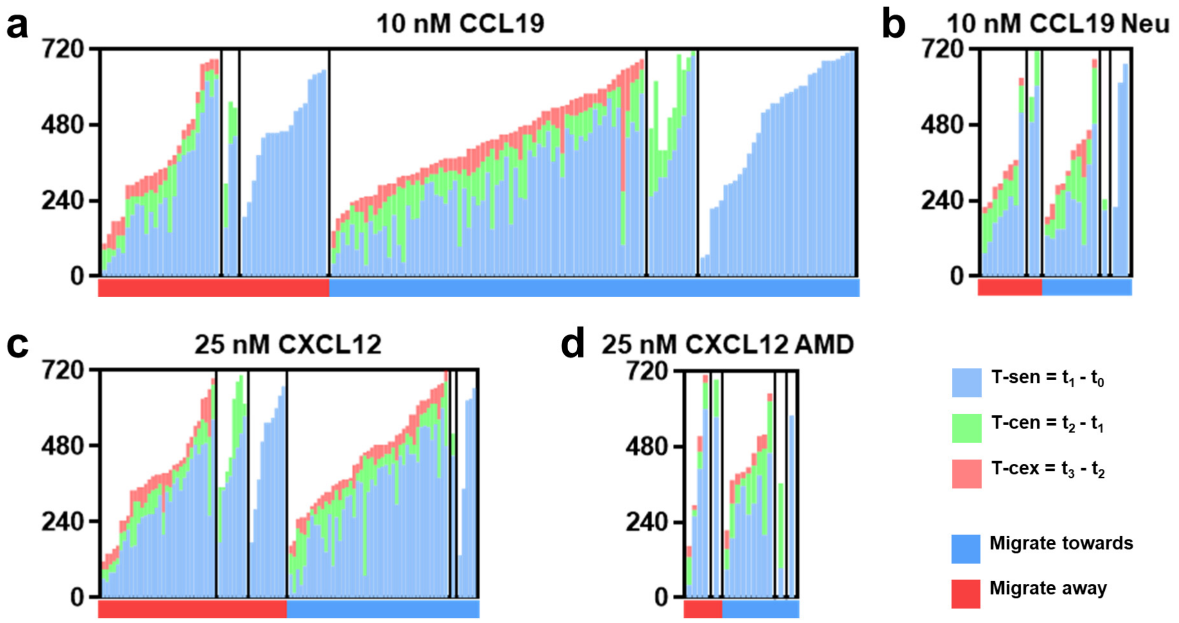

Figure S9. a,b) Migration profiles of migrating mDCs in response to 10 nM CCL19 without (a) and with (b) CCR7 neutralization. c,d) Migration profiles of migrating mDCs in response to 25 nM CXCL12 without (c) and with (d) CXCR4 antagonization. Each column represents a single migrating cell. Results represent combined data from three or two independent experiments. The total numbers of recorded cells N=523 for (a), 162 for (b), 370 for (c) and 197 for (d).

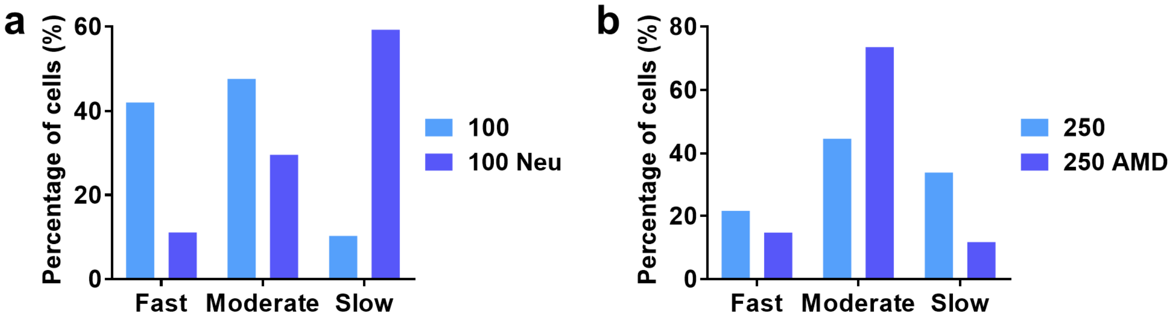

Figure S10. Changes of the percentages of fast, moderate and slow migrators in migrated-out cells towards 100 nM CCL19 with/without CCR7 neutralization by MAB197 (a) and towards 250 nM CXCL12 with/without CXCR4 antagonization by AMD3100 (b). Results represent combined data from three or two experiments.

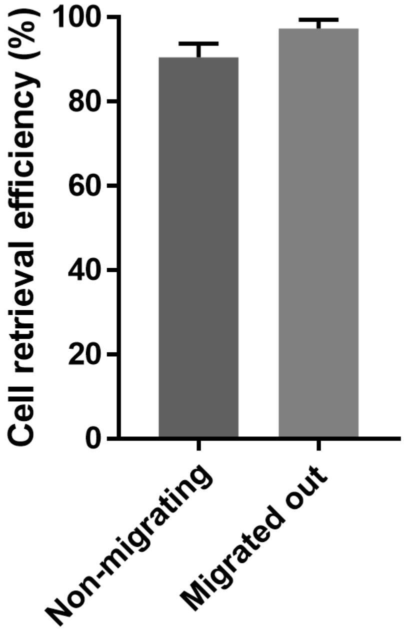

Figure S11. The mDC retrieval efficiency after BM-Chip migration assay with 50 nM CCL19. The cell numbers in the channels and in the reservoirs before and after retrieval were counted to calculate the retrieval efficiency. Results represent combined data from four independent experiments.

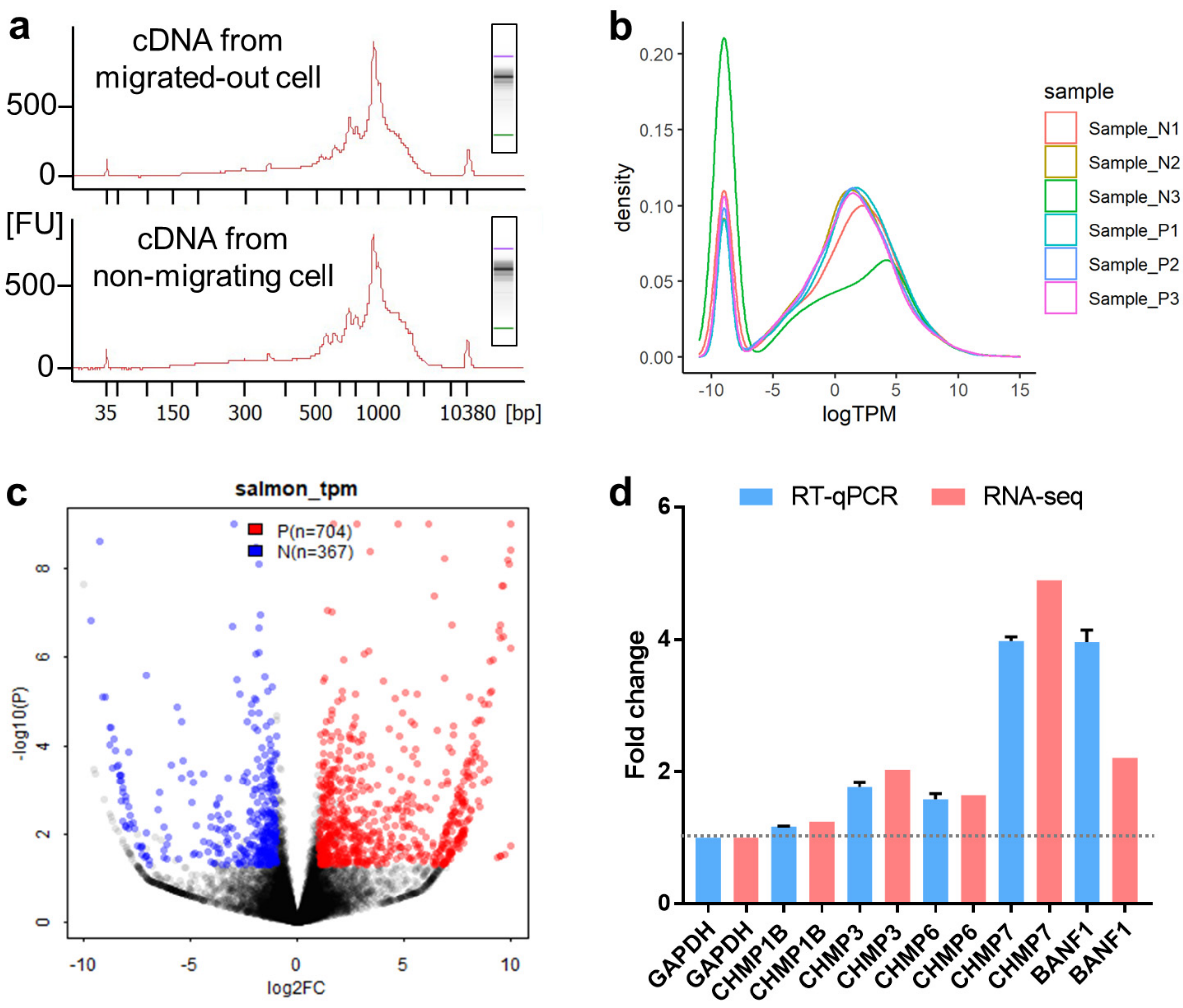

Figure S12. Evaluation of the RNA-seq data. a) Bioanalyzer traces of amplified cDNA indicate successful RNA isolation and cDNA synthesis from migrated-out cells and non-migrating cells. b) Gene expression density profile of the six samples. N1−N3 are samples of non-migrating cells and P1−P3 are samples of migrated-out cells. Sample N3 was excluded in the following differential gene expression analysis due to remarkable inconsistency with other samples. c) Volcano plot of the differentially expressed genes. P, migrated-out cells; N, non-migrating cells. d) A group of genes that are enriched in migrated-out cells were selected to validate RNA-seq data using quantitative real-time PCR. GAPDH was selected as a reference gene.

Reference
[1] P. M. Davidson, J. Sliz, P. Isermann, C. Denais, J. Lammerding, *Integr. Biol.* **2015**, *7*, 1534.
[2] C. T. Veldkamp, E. Kiermaier, S. J. Gabel-Eissens, M. L. Gillitzer, D. R. Lippner, F. A. DiSilvio, C. J. Mueller, P. L. Wantuch, G. R. Chaffee, M. W. Famiglietti, D. M. Zgoba, A. A. Bailey, Y. Bah, S. J. Engebretson, D. R. Graupner, E. R. Lackner, V. D. LaRosa, T. Medeiros, M. L. Olson, A. J. Phillips, H. Pyles, A. M. Richard, S. J. Schoeller, B. Touzeau, L. G. Williams, M. Sixt, F. C. Peterson, *Biochemistry* **2015**, *54*, 4163.
